# External Validation of Two Established Clinical Risk Scores Predicting Outcome after Local Treatment of Colorectal Liver Metastases in a Nationwide Cohort

**DOI:** 10.3390/cancers14102356

**Published:** 2022-05-10

**Authors:** Karen Bolhuis, G. Emerens Wensink, Marloes A. G. Elferink, Marinde J. G. Bond, Willemieke P. M. Dijksterhuis, Remond J. A. Fijneman, Onno W. Kranenburg, Inne H. M. Borel Rinkes, Miriam Koopman, Rutger-Jan Swijnenburg, Geraldine R. Vink, Jeroen Hagendoorn, Cornelis J. A. Punt, Sjoerd G. Elias, Jeanine M. L. Roodhart

**Affiliations:** 1 Department of Medical Oncology, Cancer Center Amsterdam, Amsterdam UMC, University of Amsterdam, 1081 HV Amsterdam, The Netherlands; k.bolhuis@amsterdamumc.nl (K.B.); w.p.dijksterhuis@amsterdamumc.nl (W.P.M.D.); 2 Department of Medical Oncology, University Medical Center Utrecht, Utrecht University, 3584 CX Utrecht, The Netherlands; g.e.wensink@umcutrecht.nl (G.E.W.); m.koopman-6@umcutrecht.nl (M.K.); g.vink@iknl.nl (G.R.V.); s.elias@umcutrecht.nl (S.G.E.); 3 Department of Research and Development, Netherlands Comprehensive Cancer Organisation (IKNL), 3511 DT Utrecht, The Netherlands; m.elferink@iknl.nl; 4 Department of Epidemiology, Julius Center for Health Sciences and Primary Care, University Medical Center Utrecht, Utrecht University, 3584 CG Utrecht, The Netherlands; m.j.g.bond-2@umcutrecht.nl (M.J.G.B.); c.j.a.punt@umcutrecht.nl (C.J.A.P.); 5 Department of Pathology, The Netherlands Cancer Institute, 1066 CX Amsterdam, The Netherlands; r.fijneman@nki.nl; 6 Utrecht Platform for Organoid Technology, University Medical Center Utrecht, Utrecht University, 3584 CX Utrecht, The Netherlands; o.kranenburg@umcutrecht.nl; 7 Department of Surgery, University Medical Center Utrecht, Utrecht University, 3584 CX Utrecht, The Netherlands; i.h.m.borelrinkes@umcutrecht.nl (I.H.M.B.R.); j.hagendoorn-3@umcutrecht.nl (J.H.); 8 Department of Surgery, Cancer Center Amsterdam, Amsterdam UMC, University of Amsterdam, 1105 AZ Amsterdam, The Netherlands; r.j.swijnenburg@amsterdamumc.nl

**Keywords:** colorectal cancer, liver metastases, prediction model, resection, survival

## Abstract

**Simple Summary:**

Patients with colorectal liver metastases (CRLM) are able to achieve long-term survival when they receive local treatment of CRLM (resection or tumor ablation). Existing clinical risk scores (CRSs) predicting prognosis of patients after resection of colorectal liver metastases were developed in highly specialized centers and thus may not function in the general population. We validated the Fong and GAME CRSs in a large population-based cohort, including two important subgroups: young/elderly and with/without perioperative chemotherapy. Both CRSs showed predictive ability. However, they were not able to discriminate preoperative risk sufficiently for clinical decision-making and, thus, require improvement.

**Abstract:**

Optimized surgical techniques and systemic therapy have increased the number of patients with colorectal liver metastases (CRLM) eligible for local treatment. To increase postoperative survival, we need to stratify patients to customize therapy. Most clinical risk scores (CRSs) which predict prognosis after CRLM resection were based on the outcome of studies in specialized centers, and this may hamper the generalizability of these CRSs in unselected populations and underrepresented subgroups. We aimed to externally validate two CRSs in a population-based cohort of patients with CRLM. A total of 1105 patients with local treatment of CRLM, diagnosed in 2015/2016, were included from a nationwide population-based database. Survival outcomes were analyzed. The Fong and more recently developed GAME CRS were externally validated, including in pre-specified subgroups (≤70/>70 years and with/without perioperative systemic therapy). The three-year DFS was 22.8%, and the median OS in the GAME risk groups (high/moderate/low) was 32.4, 46.7, and 68.1 months, respectively (*p* < 0.005). The median OS for patients with versus without perioperative therapy was 47.6 (95%CI [39.8, 56.2]) and 54.9 months (95%CI [48.8, 63.7]), respectively (*p* = 0.152), and for below/above 70 years, it was 54.9 (95%CI [49.3–64.1]) and 44.2 months (95%CI [37.1–54.3]), respectively (*p* < 0.005). The discriminative ability for OS of Fong CRS was 0.577 (95%CI [0.554, 0.601]), and for GAME, it was 0.596 (95%CI [0.572, 0.621]), and was comparable in the subgroups. In conclusion, both CRSs showed predictive ability in a population-based cohort and in predefined subgroups. However, the limited discriminative ability of these CRSs results in insufficient preoperative risk stratification for clinical decision-making.

## 1. Introduction

Approximately 30% of patients with colorectal cancer (CRC) develop liver metastases (CRLM) [1]. Currently, local treatment of CRLM (e.g., resection or tumor ablation) offers the only chance for long-term survival, with 5-year overall survival (OS) rates of up to 55% [2,3,4]. Surgical techniques continue to evolve, with two-stage resections including associating liver partition and portal vein ligation for staged hepatectomy (ALPPS); and laparoscopic liver resections, including minor/major resections, robotic hepatectomy, anatomic resections, parenchymal sparing strategies, and minimally invasive procedures for simultaneous resections of liver metastases and primary CRC [5,6]. Improved surgical procedures, more lenient resection criteria, and optimization of induction systemic therapy have increased the number of patients with CRLM that are considered technically resectable [7,8]. However, relapse after liver resection occurs in up to 75% of patients [9,10,11], and a subgroup of patients have no long-term OS benefit, due to aggressive tumor biology. This underscores the urgent need to improve risk-stratification prior to surgery [12].

An ideal clinical risk score (CRS) for these patients should identify patients with a high risk of early recurrence after surgery in order to prevent major surgery with associated risk of perioperative morbidity and mortality. Among earlier CRSs for patients with CRLM [2,13,14], the Fong score—developed in 1999 [15]—is still used most frequently to predict prognosis after liver resection [16]. The Fong CRS incorporated lymph node status, CEA value, disease-free interval (DFI), and size and number of liver metastases [15]. However, essential validation efforts of these earlier CRSs are scarce [17,18,19], especially in populations receiving modern systemic therapies, improved surgical, and ablative treatment options [2,13,14,15].

Novel CRSs [16,20,21,22,23,24] have been proposed with their own strengths and limitations, including the modified clinical score (m-CS) [20], Liverpool score [23], comprehensive evaluation of relapse risk score (CERR) [22], alternative clinical score (a-CS) [24], and the Genetic And Morphological Evaluation (GAME) score [16]. The GAME score incorporates recalibrated tumor markers such as KRAS mutational status, extrahepatic disease presence, and Tumor Burden Score (TBS). The TBS is suggested to better correlate with OS compared to separate information on the number and size of metastases [25]. The GAME score outperformed the Fong score in two single-institution patient cohorts but lacks external validation in more unselected patient cohorts.

Overall, the generalizability of these CRSs to routine care remains questionable. The scores were developed in single and/or specialized liver centers and validated in other specialized centers, potentially not reflecting results in a general population of patients with CRLM [19,26]. Furthermore, important subgroups were underrepresented in the development and validation cohorts such as elderly patients, who represent 50% of the CRC population and who are increasingly offered local liver treatment, as long-term survival can also be achieved in these patients undergoing resection of CRLM [27,28,29]. Lastly, geographical differences in treatment guidelines might influence cohort characteristics and, therefore, risk score performance. For example, the GAME score was developed and validated in the United States of America, with the majority of patients receiving perioperative systemic therapy according to local guidelines [30], while other guidelines do not recommend standard (neo)adjuvant systemic therapy [31,32].

The aim of this study was to evaluate the generalizability and clinical validity of two CRSs, the widely used Fong score and the more recent GAME score, in a nationwide population-based cohort of patients after local treatment of CRLM. Furthermore, we validated both CRSs in two pre-specified subgroups: with/without modern perioperative systemic therapy and age below/above 70 years.

## 2. Materials and Methods

### 2.1. Population-Based Cohort

All patients initially diagnosed with CRC between 1 January 2015 and 31 December 2016 and who underwent local treatment (resection and/or local ablation) for CRLM were identified in the Netherlands Cancer Registry (NCR; IRBdm20-162). The NCR is a population-based registry with clinical data of all newly diagnosed cancer patients in the Netherlands, based on notification of newly diagnosed malignancies in the Netherlands by the national automated pathological archive (PALGA [33]) or national registry of hospital discharge. PALGA comprises all patients with histologically confirmed cancer in the Netherlands. Patients with extrahepatic metastases before resection, R2 liver resections, appendix carcinoma, concomitant local liver treatments other than resection or ablation, and inadequate follow-up information were excluded. The research protocol and use of this data was approved by the Netherlands Comprehensive Cancer Organisation (IKNL). Written informed consent was not applicable according to national legislation. The study was performed in accordance with the Declaration of Helsinki.

### 2.2. Clinical Data

Pseudonymized clinical data were retrieved from the NCR and PALGA, including age, sex, American Joint Committee on Cancer (AJCC) tumor status (T-status), nodal status (N-status; N0, N1, and N2), location of primary tumor (left, right, rectum), DFI between detection of primary tumor and metastases, size and number of metastases, serum carcinoembryonic antigen (CEA) level (ug/L) prior to liver resection, type of local treatment, resection margin status (R0 was defined as a microscopically tumor free surgical margin), and RAS/BRAFV600E mutational status. TBS [25] was calculated. A major resection was defined as resection of ≥4 liver segments [34], synchronous disease as a DFI of ≤6 months [35], and perioperative systemic therapy as any systemic therapy administered within 100 days before and/or after local treatment of CRLM and initiated prior to progression of disease after resection. No distinction could be made between neo-adjuvant or induction systemic therapy in the NCR data, because intention of treatment was not registered. However, the Dutch guidelines for CRC [31] recommend not to administer perioperative systemic therapy in initially resectable CRLM contrary to the NCCN guidelines [30]. Thus, patients who have received preoperative systemic treatment are assumed to have undergone induction treatment for initially unresectable or potentially resectable CRLM. All assumptions regarding systemic treatment can be found in Supplementary Table S1.

### 2.3. Overall Survival and Disease-Free Survival

Follow-up data for recurrences were collected from medical records by trained data managers from the IKNL until May 2020, and vital status was obtained by linkage with the municipal population registry on 31 January 2021. OS was defined as the date of first CRLM resection/ablation till the date of vital status. Disease-free survival (DFS) was defined as the date of first CRLM resection/ablation till date of a DFS event, which was defined as recurrence of disease or death, whichever occurred first, or censored on last date of DFS. If the follow-up for recurrences was shorter than the follow-up for vital status, all vital status follow-up beyond the last follow-up for recurrences was discarded for assessment of DFS. All survival assumptions are included in Supplementary Table S1.

### 2.4. RAS and BRAFV600E Mutational Status

Tumor KRAS (codons 12, 13, 61, 117, and 146), NRAS (codons 12, 13, and 61) and BRAF V600E mutational status, as ascertained during routine clinical care, were retrieved from the NCR and PALGA [33]. As mutational status is generally only determined clinically if there is an indication for (palliative) systemic treatment, this information was not available for all patients. To further complement the RAS/BRAFV600E mutational status of the cohort, we aimed to sequence >170 available tumor tissues (the first 171 available of 250 requested) by Sequenom Massarray [36]. We specifically selected these 250 patients, as they had the lowest predicted chance of having a clinically assessed mutational status according to their clinicopathological profile (based on a logistic regression propensity score for mutational status with 16 clinicopathological variables). We used this strategy to improve the chance of successful multiple imputation and of accommodating the missing at random assumption (see below).

### 2.5. Statistical Analysis and Handling of Missing Data

The study population was described using standard descriptive statistics, overall, according to systemic treatment, and according to age, using median values and interquartile interval (IQI) for continuous variables and frequencies and percentages for categorical variables. Differences between systemic treatment and age groups were statistically tested by the Mann–Whitney U test or the Fisher’s Exact Test. All reported *p*-values are two-sided and *p* < 0.05 was considered statistically significant.

To handle missing data in the context of survival analysis, we performed multiple imputation by using a substantive model compatible fully conditional specification (SMC-FCS) approach [37], assuming missingness at random. The substantive model was a Cox proportional hazards model for OS which contained the following variables: T-status, N-status, KRAS mutational status, number and size of liver metastases, CEA, systemic perioperative treatment type, sidedness of the primary tumor, age, DFI, R-status, GAME CRS, Fong CRS, and TBS (with the last 3 being passively imputed in the model). We generated 53 imputed datasets based on the percentage of patients with at least one missing key variable.

Kaplan–Meier (KM) curves were created for OS and DFS. Using the multiple imputed dataset, pooled statistics were obtained by using Rubin’s rules, including number at risk for given time points, log-rank subgroup comparison, and survival estimates with confidence intervals (using log–log transformation prior to pooling for the latter two) [38,39].

### 2.6. External Validation of CRSs

The GAME [16] and Fong score [15] were externally validated following the TRIPOD guidelines sections pertinent to external validation studies [40]. Predictive performances were assessed by measures of calibration and discrimination. Calibration was evaluated by digitizing the originally published KM curves of scores by WebPlotDigitizer version 4.4 [41] and plotted together with the observed KM curves of the NCR cohort. Discrimination was calculated by Harrell’s concordance index (C-index) across each imputed dataset and pooled by using Rubin’s rules. The C-index reflects the ability of the model to differentiate between patients who do and do not experience an event, with 0.5 representing a model without any discriminatory ability beyond chance and 1 perfect discrimination [42].

Patients were assigned to low, moderate, or high CRS risk categories, as described previously [16]: low risk, 0–1 points; moderate risk, 2–3 points, and high risk, 4 or more points, with similar allocation for the GAME and Fong CRS points.

To analyze the overlap in risk groups following the two CRSs, a contingency table and heatmap were made. External validation was repeated for the following subgroups: perioperative systemic therapy (yes/no) and age (≤70/>70 years). An analysis was performed in IBM SPSS Statistics (Version 26) and R (Version 4.0.3 for Windows) with the mice (3.13.0), smcfcs (1.5.0), survival (3.2-7), and rms (6.2-0) packages.

## 3. Results

### 3.1. Patient Characteristics

**Figure 1:**
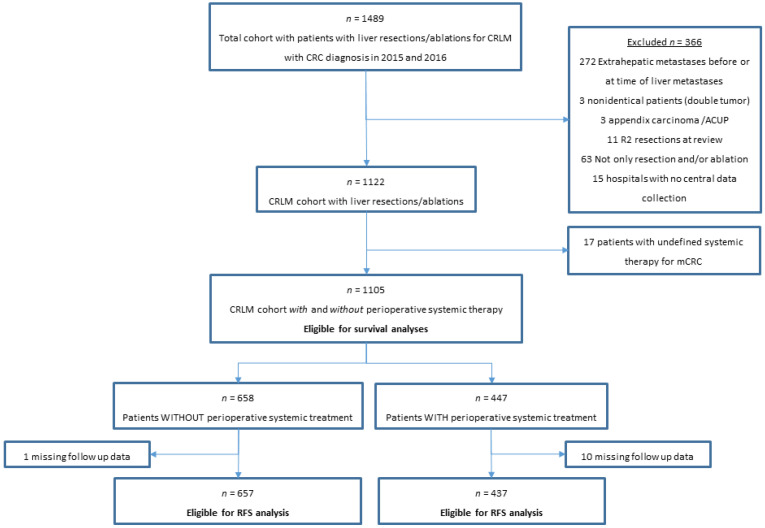
Flowchart of population-based NCR patients with local liver treatment for CRLM included in the study. Abbreviations: ACUP, adenocarcinoma with unknown primary; CRLM, colorectal liver metastases; mCRC, metastatic colorectal cancer; RFS, recurrence-free survival.

A total of 1105 patients fulfilled the eligibility criteria (1105/1489) (Figure 1). The cohort comprised 447 (40%) patients with and 658 (60%) patients without perioperative systemic therapy and 759 (69%) patients ≤ 70 and 346 (31%) patients > 70 years. Among patients with perioperative systemic treatment, 334 (75%) received preoperative-only, 54 (12%) postoperative-only, and 59 (13%) received pre- and postoperative systemic treatment. The patient characteristics are displayed in Table 1. The median age of patients was 66 years, with 690 (62%) males, and 823 (75%) patients had synchronous disease. (Table 1). Patients were treated in a total of 39 hospitals, with 45% of patients treated in academic, 44% in teaching, and 11% in regional hospitals.

### 3.2. Follow-Up and OS and DFS Outcomes in Total Cohort

**Figure 2:**
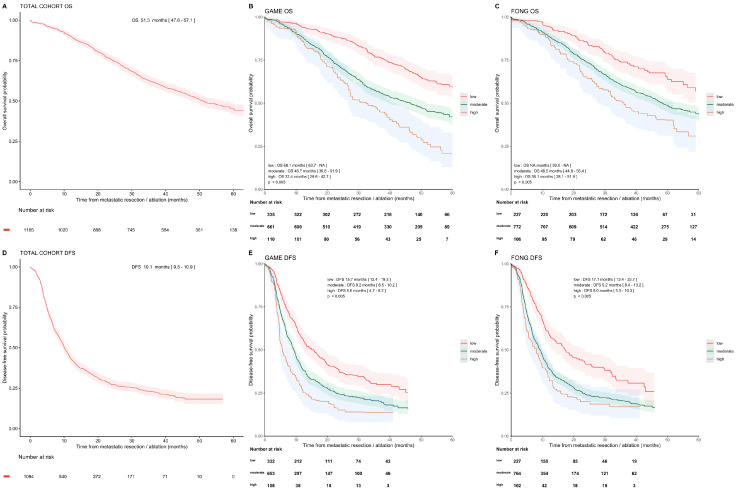
Overall survival and disease-free survival in cohort and subgroups. Kaplan–Meier analysis showing OS and DFS curves and 95% confidence intervals of the total cohort and for the risk categories following the GAME and Fong scores. OS for total cohort (**A**), and OS for GAME CRS risk groups (**B**), OS for Fong CRS risk groups (**C**). DFS for total cohort (**D**), DFS for GAME CRS risk categories (**E**), and DFS for Fong CRS risk categories (**F**).

The median follow-up for OS and DFS was 53.7 and 35.0 months, with 556 (50%) and 807 (73%) documented events, respectively. The median OS was 51.3 months (95%CI [47.6, 57.1]), and the median DFS was 10.1 months (95%CI [9.5, 10.9], Figure 2). One-, three-, and five-year OS rates were 89.9% (95%CI [88.2, 91.7]), 61.8% (95%CI [59.0, 64.8]), and 44.9% (95%CI [41.6, 48.4]), whereas the one- and three-year DFS rates were 43.1% (95%CI [40.2, 46.1]) and 22.8% (95%CI [20.2, 25.8]).

### 3.3. External Validation of GAME and Fong CRSs in Total Cohort

The study characteristics of the development cohorts of the GAME and Fong CRSs were compared to the NCR validation cohort (Table 2). The percentage of patients with adjuvant systemic therapy was 71% in the GAME cohort compared to 6% in our NCR cohort; the percentage was not reported for the Fong cohort. In the development cohort of GAME CRS, patients with extrahepatic disease were included, while these patients were excluded in the Fong cohort and the NCR cohort.

The OS and DFS of the high, moderate, and low GAME and Fong risk groups are presented in Figure 2. The OS and DFS gradually decrease per point increase for both the GAME and Fong score (Supplementary Figure S1).

By analyzing the calibration of the CRSs, we see that the original survival curves of low- and high-risk GAME groups overlapped well with the corresponding curves in our validation cohort. The GAME moderate-risk group, however, showed a shorter median OS compared to the development cohort, 46.7 versus 60 months (Supplementary Figure S2).

Overall, the discriminative ability of the GAME versus the Fong score, as measured by the Harrell’s C-index for OS, was weak, 0.596 (95%CI [0.572, 0.621]) versus 0.577 (95%CI [0.554, 0.601]), respectively. The C-indexes of OS and DFS and the pooled survival estimates per risk group and per given time-point are depicted in Table 3.

In a head-to-head comparison of the GAME and Fong CRSs, 730 patients (66.0%) were categorized in the same risk group in both prediction models. Only three patients (0.3%) showed major discordance (categorized as GAME high risk and Fong low risk). The frequency distributions among the Fong/GAME combination risk categories and corresponding survival curves are shown in Supplementary Figure S3.

### 3.4. External Validation of GAME and Fong CRSs in Pre-Specified Subgroups

#### 3.4.1. With and without Perioperative Systemic Therapy

**Figure 3:**
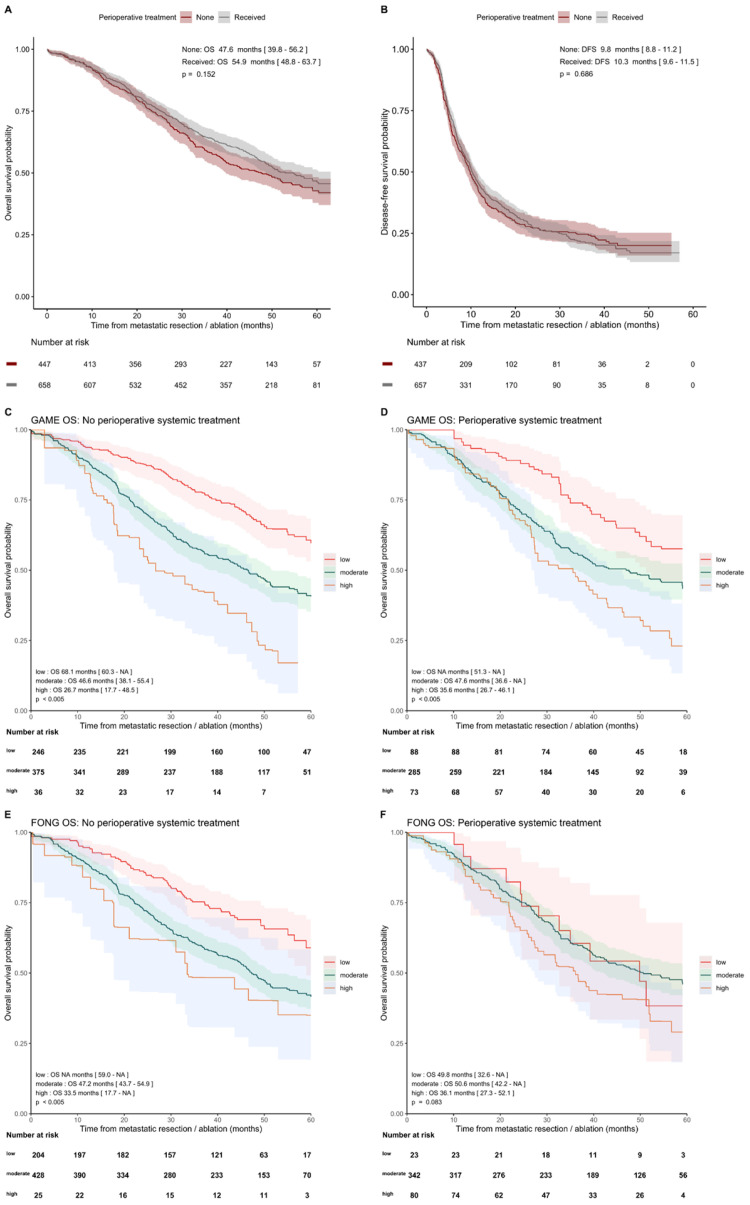
Kaplan–Meier analysis showing OS and DFS curves in patients with and without perioperative systemic therapy for the GAME and Fong risk categories. (**A**) OS and (**B**) DFS in patients with and without perioperative systemic therapy. OS outcomes of the GAME risk categories were analyzed in the subgroup without (**C**) and with perioperative systemic therapy (**D**) and OS outcomes of the Fong risk categories in subgroups of patients without (**E**) and with perioperative systemic therapy (**F**).

Although prognostic patient characteristics were unfavorable for patients with perioperative systemic therapy (Table 1), comparable survival outcomes were found in patients with and without perioperative systemic treatment, with a median OS of 47.6 (95%CI [39.8, 56.2]) and 54.9 months (95%CI [48.8, 63.7]; *p* = 0.152) and median DFS of 9.8 (95%CI [8.8, 11.2]) versus 10.3 months (95%CI [9.6, 11.5]; *p* = 0.686), respectively. GAME high-risk patients with perioperative systemic therapy had a longer median OS of 35.6 (95%CI [26.7, 46.1]) compared to patients without systemic therapy (median OS 26.7 months, 95%CI [17.7, 48.5]) (Figure 3) and a longer median DFS of 5.9 months (95%CI [4.8, 10.9]) versus 4.6 months (95%CI [3.9, 10.0]) (Supplementary Figure S4). A survival advantage for patients receiving perioperative systemic therapy was not evident in the low- and moderate-risk groups (Supplementary Figure S4). The GAME C-index for patients with and without peri-operative systemic therapy for OS was 0.590 (95%CI [0.554, 0.626]) versus 0.602 (95%CI [0.569, 0.635]), and the Fong C-index was 0.556 (95%CI [0.519, 0.594]) versus 0.593 (95%CI [0.563, 0.624]), respectively (Supplementary Table S2).

#### 3.4.2. Age ≤ 70 Years and >70 Years

**Figure 4:**
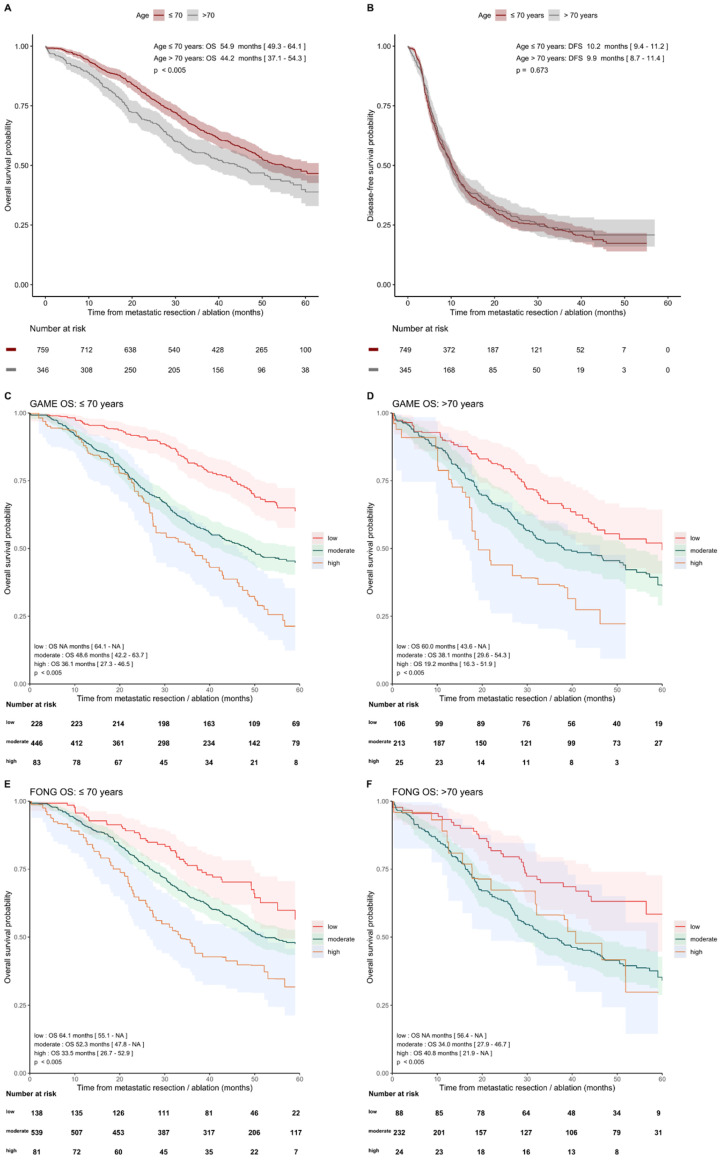
Kaplan–Meier analysis showing OS and DFS curves in patients with age ≤70 years and >70 years for the GAME and Fong risk categories. (**A**) OS and (**B**) DFS in patients with age ≤70 years and >70 years. Subsequently, the OS outcomes of the GAME risk categories were analyzed in the subgroup ≤70 years (**C**) and >70 years (**D**) and of the Fong risk categories in subgroups of patients ≤ 70 years (**E**) and >70 years (**F**).

The median OS of 54.9 months (95% CI [49.3–64.1]) was higher in patients ≤ 70 years compared to 44.2 months (95% CI [37.1–4.3]) in patients > 70 years (*p* < 0.005). The median DFS was similar for 10.2 months (95%CI [9.4, 11.2]) versus 9.9 months (95%CI [8.7, 11.4]; *p* = 0.673) (Figure 4). The discriminative ability for OS of GAME CRS and Fong CRS was comparable in both age groups, with GAME C-indexes of 0.613 (95%CI [0.584, 0.642]) and 0.575 (95%CI [0.531, 0.618]) and Fong C-indexes of 0.583 (95%CI [0.554, 0.612]) and 0.589 (95%CI [0.548, 0.630]), respectively, for below/above 70 years. The C-indexes for one-, three-, and five-year OS and DFS of GAME versus Fong in predefined subgroups are shown in Supplementary Table S3.

## 4. Discussion

In this study, we externally validated and compared two established CRSs, the GAME and Fong score, for their ability to predict OS and DFS after resection of CRLM in the modern era in a real-life population-based cohort and in two pre-specified subgroups. Both CRSs showed predictive ability with a better performance of the GAME as compared to the traditional Fong CRS. The external validation in subgroups of both CRSs showed a comparable performance in patients with and without perioperative systemic therapy and in patients ≤ 70 and >70 years. However, the overall predictive performance remained suboptimal, with a high prognostic uncertainty which limits its utility in clinical decision-making.

The GAME score was originally validated in a cohort of patients from specialized institutes, while the Fong score was not validated in the original paper. This could hamper their generalizability to real-life patients. In our real-life cohort, we found a similar C-index for the GAME and Fong score for OS as compared to the C-indexes published by Margonis et al. [16]. In our cohort, the GAME score outperformed the traditional Fong score. Both CRSs show discriminatory ability, but since C-indexes are 0.6 at most, a significant level of prognostic uncertainty remains. Furthermore, 25% of patients identified as “high-risk” according to the GAME score did achieve long-term survival, which exceeded five years, and this rate was even higher in the Fong high-risk group. This signifies that, although these CRSs might be used for risk counselling and managing expectations of patients, they cannot be used for clinical decision-making to select high-risk patients for whom surgery should be avoided or low-risk patient for whom extensive surgery may be justified.

To improve the prognostic performance of a CRS, categorizing variables should be avoided, and simplification of the CRS by a point system or classification in risk groups is not always desirable. While this strategy is performed to gain usability, it also results in the loss of information. One way to ensure model usability, while avoiding simplification, is to use a web calculator, along with a prediction model, which could be incorporated into electronic patient management systems for clinicians and patients [24].

Evolving molecular research results in newly recognized tumor biomolecular prognostic markers and shows the heterogeneity of CRLM. The GAME CRS incorporated KRAS codon 12, 13, and 61 only. However, BRAFV600E mutation is recognized to be a strong prognostic factor, as well which negatively influences post-resection survival outcomes. Other molecular markers are proposed as prognostic markers too, such as mutations in the SMAD family, TP53, and PIK3CA. In future practice, by incorporating novel biomarkers and integrating molecular subtypes, clinical risk stratification may be improved [43].

Other recently published CRSs were not externally validated on our cohort for various reasons. The m-CS [20] simplified the traditional CRS and replaced two risk factors by RAS mutational status, and the Liverpool score [23] did not incorporate RAS mutation status in its CRS, which is recognized to be the most promising prognostic factor in patients with CRLM [44,45,46]. The Chinese CERR [22] included two variables (serum CA 19.9 and bilobar liver distribution of metastatic disease) which were not available in our cohort. For the a-CS [24], discrepancies in the published survival outcomes and the web-based calculation tool of the a-CS complicate external validation.

When comparing the OS of our population-based cohort with the original GAME cohort, we found a lower median OS in our GAME moderate-risk group. Survival was similar in the GAME low- and high-risk groups. The difference in the moderate-risk group could potentially be influenced by treatment setting. The GAME cohort concerned a selected population treated in a tertiary center with potentially more (experimental) treatment options available, in contrast to our population-based cohort. We did not observe a survival difference between the moderate-risk groups in the subgroups with or without systemic treatment. Therefore, it is unlikely that the greater proportion of systemic therapy administered to the GAME cohort explains the survival differences in the moderate-risk group in our cohort versus the GAME cohort.

Furthermore, as our cohort consists of patients with and without perioperative systemic therapy, we could demonstrate additional interesting survival outcomes. Patients who received perioperative systemic therapy were found to have more prognostic unfavorable characteristics, while the median OS was similar in patients with and without perioperative systemic therapy. This could imply that, in these patients, systemic therapy compensates for the more unfavorable characteristics. This is supported by the findings that patients in the high-risk CRS groups showed a longer median OS and DFS in the subgroup with versus without systemic therapy. Our results are consistent with studies suggesting that high-risk patients with CRLM could benefit from (neo)-adjuvant therapy [9,47,48,49] and is supported by the negative results of the EORTC 40983 [50] study and the JCOG0603 study [51] for perioperative systemic treatment in, respectively, patients with low-risk disease with <4 CRLM and unselected patients with CRLM. Since the results of the treatment groups are based on retrospective data, this should be confirmed in prospective trials, randomizing high-risk CRLM patients between (neo-)adjuvant therapy or not. However, conducting a study such as this one has proved to be challenging [52].

Another interesting finding is the OS difference in favor of patients ≤ 70 compared to >70 years after resection. Since it did not concern disease-specific but overall survival, other factors, such as comorbidity, might have influenced the OS in this group. This is supported by the result that DFS did not differ between these two subgroups. This OS difference should therefore not be used as an argument against liver resection in patients above 70 years.

The external validation of the CRSs in this study met the TRIPOD guidelines’ methodological criteria [40]. Additional strengths include validation of CRS in a real-life population-based cohort which is representative of the whole CRLM population and the near-complete follow-up. Furthermore, the proportion of missing RAS/BRAF mutational status was low, and this was achieved by additional mutational analysis. Selection bias was avoided in correction for missing data by including propensity score matching to identify patients for additional mutational analysis and by using multiple imputation. One limitation of this study is that the patients in our cohort were selected based on primary tumor diagnosis in 2015 and 2016. Thus, our cohort does not include patients with metachronous disease with a long DFI [53]. In addition, selection and information bias is unavoidable given the retrospective nature of the study, although we believe we minimized bias by using a population-based cohort and by handling missing data by multiple imputation. For the validation of the GAME score, mutation status as risk factor was scored by the detection of KRAS codon 12, 13, and 61 mutations, meaning that other RAS mutations were ignored to meet the exact GAME criteria, as proposed by Margonis et al. Lastly, the GAME score incorporated patients with extrahepatic disease as a risk factor. As these patients were excluded from our study, the GAME high-risk groups in our validation cohort did not include patients with a maximum of five risk factors.

## 5. Conclusions

Two established CRSs, Fong and GAME, to predict outcome after CRLM resection were compared and externally validated in a real-life population-based cohort of patients with local treatment of CRLM, regardless of age or the administration of perioperative systemic therapy. Both CRSs showed predictive ability in the real-life cohort, with a better performance of the GAME as compared to the traditional Fong CRS. Although the novel CRS (GAME) outperformed the traditional CRS, the suboptimal predictive value of both CRSs limits the clinical utility of the CRSs. Surgical innovations increase the number of CRLM patients assessed as technically resectable, but high recurrence rates persist, and a significant group of patients has no long-term survival benefit of CRLM resection. Thus, there is still an unmet clinical need for a CRS with high discriminative ability that allows for a better stratification and counselling of patients before surgery and perioperative therapy in order to personalize therapy.

## Figures and Tables

**Table 1:** Characteristics of total NCR cohort and patients with and without systemic therapy and below or above 70 years.

		NCR Cohort (*n* = 1105)	Patients without Systemic Therapy (*n* = 658)	Patients withSystemic Therapy (*n* = 447)	*p*-Value	Patients ≤ 70 Years (*n* = 759)	Patients > 70 Years (*n* = 346)	*p*-Value
Age Median (IQI)		66 (59–72)	68 (61–74)	63 (56–70)	< 0.001	62 (56–66)	75 (72–78)	<0.001
Sex					0.60			<0.008
	Male	690 (62)	415 (63)	275 (62)		454 (60)	236 (68)	
	Female	415 (38)	243 (37)	172 (39)		305 (40)	110 (32)	
Side primary tumor					0.92			0.09
	Right	261 (24)	154 (23)	107 (24)		167 (22)	94 (27)	
	Left	473 (43)	285 (43)	188 (42)		324 (43)	149 (43)	
	Rectum	371 (34)	219 (33)	152 (34)		268 (35)	103 (30)	
Chemoradiotherapy primary tumor					<0.001			0.67
	No	977 (88)	556 (85)	421 (94)		669 (88)	308 (89)	
	Yes	128 (12)	102 (15)	26 (6)		90 (12)	38 (11)	
T-status primary tumor					0.01			0.97
	1	27 (3)	21 (3)	6 (1)		19 (3)	8 (2)	
	2	128 (12)	86 (13)	42 (10)		89 (12)	39 (11)	
	3	757 (69)	455 (69)	302 (69)		516 (69)	241 (70)	
	4	185 (17)	96 (15)	89 (20)		129 (17)	56 (16)	
	Missing	8 (-)	0 (-)	8 (-)		6 (-)	2 (-)	
Nodal status primary tumor					0.22			0.71
	N0	408 (37)	257 (39)	151 (34)		277 (37)	131 (38)	
	N1	389 (35)	224 (34)	165 (37)		265 (35)	124 (36)	
	N2	306(28)	177 (27)	129 (29)		216 (29)	90 (26)	
	Missing	2 (-)	0 (-)	2 (-)		1 (-)	1 (-)	
Stage of disease at diagnosis					< 0.001			0.40
	I	25 (2)	20 (3)	5 (1)		16 (2)	9 (3)	
	II	102 (9)	87 (13)	15 (3)		65 (9)	37 (11)	
	III	187 (17)	162 (25)	25 (6)		123 (16)	64 (19)	
	IV	791 (72)	389 (59)	402 (90)		555 (73)	236 (68)	
Differentiation grade of CRC					0.12			0.77
	Low	17 (2)	7 (1)	10 (3)		13 (2)	4 (1)	
	Intermediate	936 (92)	577 (93)	359 (90)		642 (92)	294 (92)	
	High	68 (7)	37 (6)	31 (8)		46 (7)	22 (7)	
	Missing	84 (-)	37 (-)	47 (11)		58 (-)	26 (-)	
Time to metastases					<0.001			0.20
	Synchronous	823 (75)	412 (63)	411 (92)		574 (76)	249 (72)	
	Metachronous	282 (25)	246 (37)	36 (8)		185 (24)	97 (28)	
Number of liver metastases					<0.001			<0.001
	Median (IQI)	2 (1–4)	1 (1–3)	3 (2–6)		2 (1–4)	2 (1–3)	
	Missing	42	19	23		33	9	
CEA level					<0.001			0.90
	Median (IQI)	9 (3.4–36)	6.3 (3.0–21)	14 (4.4–74)		18 (4.0–413)	17 (4.7–168)	
	Unknown	231	180	51		160	71	
Size largest liver metastasis, mm					0.002			0.30
	Median (IQI)	25 (16–36)	23 (16–35)	27 (16–45)		25 (15–45)	26 (18–42)	
	Missing	86	45	41		58	28	
Type of surgery					<0.001			0.34
	Wedge/segment resection only	589 (53)	416 (63)	173 (39)		400 (53)	189 (55)	
	Local ablative therapy only	95 (9)	63 (9)	32 (7)		59 (8)	36 (10)	
	Wedge/segment and local ablative therapy	189 (17)	90 (14)	99 (22)		134 (18)	55 (16)	
	Hemihepatectomy with/without ablation/wedge (major resection)	232 (21)	89 (14)	143 (32)		166 (22)	66 (19)	
One- or two-stage					<0.001			0.84
	1-stage	1042 (94)	643 (98)	399 (89)		715 (94)	327 (95)	
	2-stage	63 (6)	15 (2)	48 (11)		44 (6)	19 (6)	
R-status					0.07			
	R0	866 (78)	521 (79)	345 (77)				0.37
	R1	143 (13)	74 (11)	69 (15)		598 (79)	268 (78)	
	Unknown because RFA/MWA	96 (9)	63 (10)	33 (7)		101 (13)	42 (12)	
Tumor mutational status					0.36			0.93
	*RAS* mutation	362 (51)	221 (53)	141 (48)		247 (50)	115 (52)	
	*BRAF^V600E^* mutation	19 (3)	10 (2)	9 (3)		13 (3)	6 (3)	
	*RAS and BRAF^V600E^* wildtype	335 (47)	188 (45)	147 (50)		233 (47)	102 (46)	
	Missing (*RAS* and/or *BRAF* status)	389 (-)	239 (-)	150 (-)		266 (-)	123 (-)	

Abbreviations: CEA, Carcinoembryonic Antigen; CRC, colorectal cancer; IQI, Interquartile range; MMR, mismatch repair; NCR, Netherlands cancer registry.

**Table 2:** Characteristics of original Fong and GAME CRS cohorts compared to Dutch NCR cohort used for external validation.

	GAME	Fong	NCR
Number of patients (design/validation)	502/747	1001/-	-/1105
Country (design/validation)	USA/USA	USA/-	-/Dutch
Study design	Single center	Single center	Nation-wide multicenter
Patients with liver-only metastases, %	90	100	100
Handling of missing data	Patients excluded with *KRAS* status missing	NR	No patients excluded based on missing data
Available mutation status	*KRAS* codon 12, 13, and 61	-	*RAS*/*BRAF*
Primary endpoint	OS	OS	OS
Preoperative systemic therapy, %	67	NR	55
Adjuvant systemic therapy, %	71	NR	6
DFI < 12 months, %	74	49	84
Factors included in CRS, (points)	Nodal status (1) CEA > 20 (1) TBS < 9 (1) TBS ≤ 9 (2) *KRAS* mutation (1) Extrahepatic disease (2)	Nodal status (1) CEA > 200 (1) DFI < 1year (1) >1 Liver tumor (1) Largest tumor > 5 cm (1)	-

Abbreviations: CEA, carcinoembryonic antigen; cm, centimeters; CRS, clinical risk score; DFI, disease-free interval; GAME, genetic and morphological evaluation score; NCR, Netherlands cancer registry; NR, not reported; OS, overall survival; TBS, tumor burden score; USA, United States of America.

**Table 3:** Pooled Harrell’s concordance index with 95% confidence intervals for 1-, 3-, and 5-year overall survival and disease-free survival outcomes for GAME and Fong risk scores and survival estimates at 1-, 3-, and 5 years for low-, moderate-, and high-risk groups according to GAME and Fong prediction model.

	GAME Score	Survival Estimates GAME Risk Categories			Fong Score	Survival Estimates Fong Risk Categories		
	C-Index [95% CI]	Low (%)	Moderate (%)	High (%)	C-Index [95% CI]	Low (%)	Moderate (%)	High (%)
OS								
1-year	0.583 [0.531–0.636]	94	88	86	0.570 [0.521–0.619]	95	89	87
3-year	0.600 [0.573–0.627]	77	57	47	0.578 [0.552–0.604]	74	60	50
5-year	0.597 [0.573–0.621]	50	42	21	0.577 [0.554–0.601]	57	40	31
DFS								
1-year	0.585 [0.561–0.608]	57	39	27	0.586 [0.564–0.608]	60	39	34
3-year	0.579 [0.557–0.600]	30	21	14	0.581 [0.561–0.602]	32	20	17

Abbreviations; C-index, concordance index; DFS, disease-free survival; OS, overall survival.

## Data Availability

The datasets generated during and analyzed during the current study are not publicly available, due to the Netherlands Cancer Registry regulations, but they are available from the corresponding author or Netherlands Cancer Registry upon reasonable request.

## Supplementary Materials

The following supporting information can be downloaded at https://www.mdpi.com/article/10.3390/cancers14102356/s1. Supplementary Table S1. Assumptions regarding baseline characteristics, systemic treatment, local treatment, and survival outcomes. Supplementary Figure S1. Kaplan–Meier analysis showing overall survival and disease-free survival curves of the total cohort in scores following the GAME clinical risk score (A,B) or the Fong clinical risk score (C,D). Supplementary Figure S2. Calibration of the expected and observed survival outcomes in the NCR cohort of the risk groups (low, moderate, and high) according to (A) GAME and (B) Fong prediction model. Supplementary Figure S3. A combined figure containing, on the left, a contingency table showing the frequency distribution of patients among the risk categories (low, moderate, and high) following the Fong and GAME prediction model, as well as their corresponding 3-year survival rate estimate, which is also indicated by the heat map for each category. The corresponding survival curves for the groups are displayed in the KM plot in the figure on the right. Supplementary Figure S4. Kaplan–Meier analysis showing disease-free survival curves for GAME risk groups for patients without (A) and with (B) perioperative systemic therapy and for age groups ≤ 70 years (E) and > 70 years (F). The DFS outcomes of the Fong risk groups are shown for patients without (C) and with (D) perioperative systemic therapy and for age groups ≤ 70 years (G) and > 70 years (H). Supplementary Table S2. Pooled Harrell’s concordance index with 95% confidence intervals for 1- and 3-year overall survival and disease-free survival outcomes for GAME and Fong risk scores in subgroups without and with perioperative systemic therapy. Supplementary Table S3. Pooled Harrell’s concordance index with 95% confidence intervals for 1- and 3-year overall survival and disease-free survival outcomes for GAME and Fong risk scores in subgroups of ≤70 years and >70 years.
