# Supplementary material for: External Validation of Two Established Clinical Risk Scores Predicting Outcome after Local Treatment of Colorectal Liver Metastases in a Nationwide Cohort
Source: Cancers (Basel). 2022 May 10;14(10):2356. doi: 10.3390/cancers14102356 (PMC9139295; doi:10.3390/cancers14102356)
Supplement: Supplementary file 1 [file cancers-14-02356-s001.zip › cancers-1682282-supplementary.pdf]

Supplementary Materials

External Validation of Two Established Clinical Risk Scores Predicting Outcome After Local Treatment of Colorectal Liver Metastases in a Nationwide Cohort

Supplementary Table S1. Assumptions regarding baseline characteristics, systemic treatment, local treatment and survival outcomes

**Assumptions regarding baseline characteristics:**	
-	RAS and BRAF mutation are considered mutual exclusive, therefore patients with RAS mutations or BRAF mutations, were assumed to have BRAF wildtype or RAS wildtype status, respectively.
-	Primary tumor nodal status was defined primarily on pathologic N-stadium. When pN stage was missing, cN stage (radiological) was used.
-	If number of metastases was not given and code 77 was used (accounting for diffuse metastatic disease in the liver) then number of metastases was scored as 20.
**Assumptions regarding systemic treatment regimens and strategies:**	
-	Systemic treatment includes both chemotherapy and/or targeted therapy.
-	A combination regimen is defined as all systemic agents starting within 4 weeks after start of the first agent and started before progression of disease.
-	If bevacizumab was started more than 4 weeks after the start of the first agent but before stop of this agent and before progression of disease, we assume bevacizumab was part of this combination regimen.
-	If a treatment line continues despite of progression, e.g., in case of reintroduction of the same or an equivalent regimen after a therapy break and detected progression, we regard this as continuation of the same treatment line.
-	If oxaliplatin only is registered, we assume this was part of a capecitabine and oxaliplatin (CapOx) regimen of which capecitabine was not registered, so we add capecitabine. We assume this is due to a registration error, in which the administration of capecitabine has not been noticed by the data manager since it is registered differently as oral medication.
-	Systemic therapy was considered adjuvant systemic therapy for primary tumor when started < 12 weeks after resection of primary tumor and started before diagnosis of metastases in patients with metachronous disease.
-	Capecitabine monotherapy was considered radiosensitizer for primary tumor when started before primary tumor resection and before diagnosis of metastases and with notification to have received chemoradiotherapy.
-	Systemic therapy was considered pre-operative therapy (neo-adjuvant or induction) before liver resection when the therapy ended within 120 days before liver resection. Adjuvant therapy after resection of primary tumor or chemotherapy as radiosensitizer was excluded.
-	Systemic therapy was considered adjuvant therapy after liver resection when the therapy started within 120 days after liver resection. Chemotherapy as radiosensitizer was excluded.
-	Systemic therapy was considered peri-operative therapy of liver resection when the systemic therapy was given < 120 days before and < 120 days after liver resection
-	When systemic therapy was given between two liver procedures before progression of disease, the first liver procedure was considered as staging procedure and systemic therapy was considered as pre-operative systemic therapy (neo-adjuvant or induction) for surgery 2
-	A treatment line is defined as systemic therapy (monotherapy or combination regimen) administered at the same time until suspension, regardless of reason for discontinuation.
-	Treatment is considered as next line if an agent of a new drug group is started that is not applied in the previous systemic treatment regimen.

- If the same or an equivalent systemic treatment regimen is (re)started, this is considered continuation of the same treatment line, e.g., CapOx to 5-FU/oxaliplatin (FOLFOX).

Assumptions regarding local treatment

- Local treatments are categorized as follows:
  - 1 stage (1 procedure)
  - 2-stage (2 procedures < 120 days apart)
- R-status: when two stage procedure and first procedure was R2 resection and second procedure was R1/R0 resection than 2-stage resection considered as R-status of last procedure.
- when 2-stage resection and one procedure was R1 resection and other local treatment was R0 resection than considered as R1 resection.

Table continued on next page.

Assumptions regarding progression of disease and survival:

- Date of new episode is considered as time of progression.
- When disease progression is documented < 14 days of liver resection we assume this was part of the liver resection and first new episode is considered as time of progression.
- Disease-free survival is calculated from date of first liver procedure to date of progression. In case of 2-stage resection, DFS is calculated from last liver procedure.
- If no recurrence is registered:
  - If end of follow up is registered and reason end of follow up is: death, then date of death is registered as event of DFS;
  - If end of follow up is registered and reason end of follow up is other than death then DFS is censored on date of end of follow up;
  - If no date of end of follow up is registered then DFS is censored on date of last visit;
  - If none of these dates are registered then DFS is documented as missing.
- Lymph node metastases registered as abdominal lymph nodes at time of first liver metastases were considered extrahepatic disease and as so classified as not-liver only disease.
- Overall survival (OS) after resection was defined as date of first resection till date of last documented vital status as documented by the municipal population registry. In case of 2-stage resection, OS is calculated from date of last liver procedure
  - If the documented date of disease-free survival is after date of documented survival than the date of disease-free survival is date of last survival
- Patients who did not die are censored on the date last known to be alive in the municipal population registry.

Supplementary Figure S1. Kaplan-Meier analysis showing overall survival and disease-free survival curves of the total cohort in scores following the GAME clinical risk score (A and B) or the Fong clinical risk score (C and D).

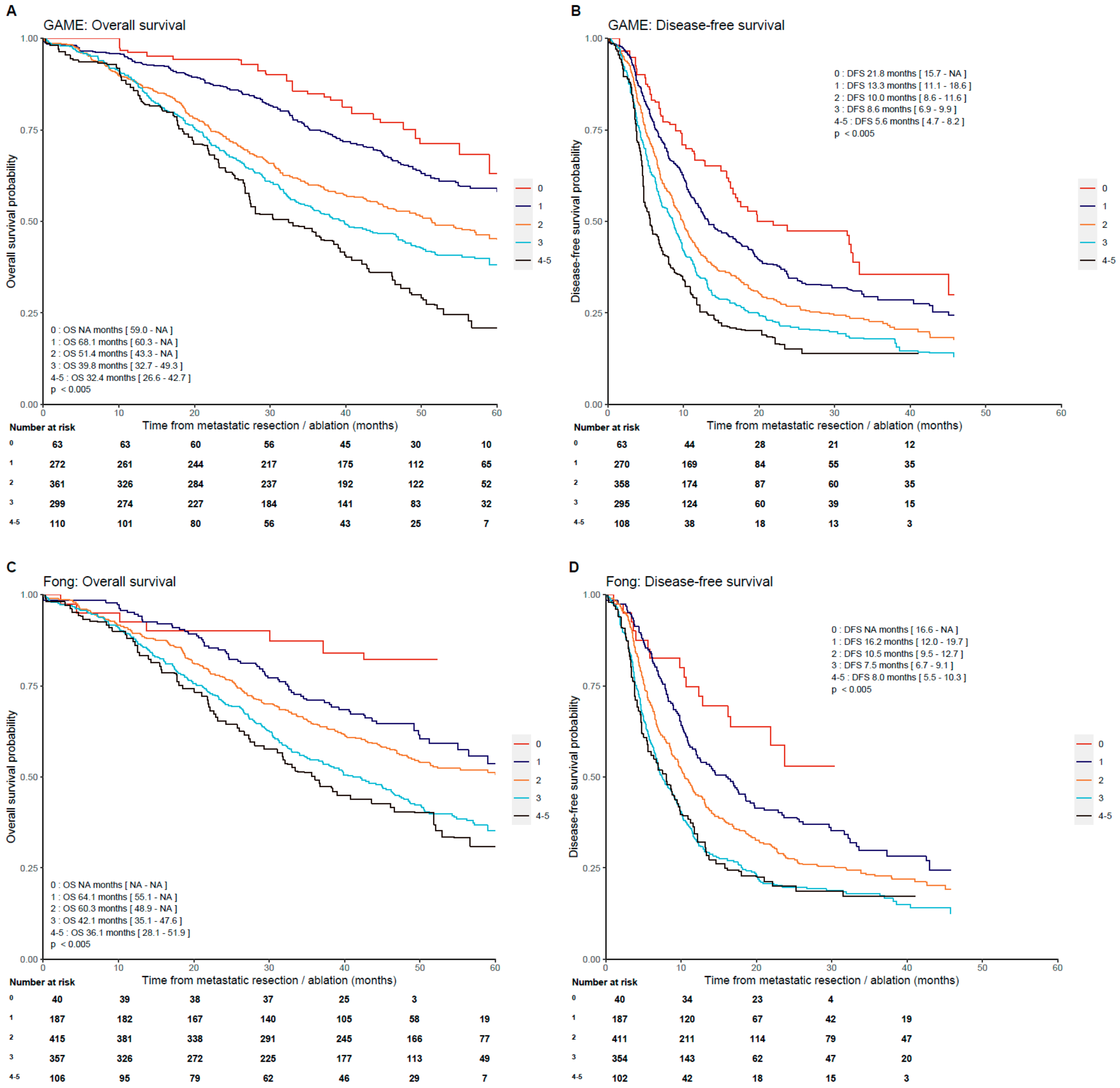

Supplementary Figure S2. Calibration of the expected and observed survival outcomes in the NCR cohort of the risk groups (low, moderate and high) according to: A) GAME and B) Fong prediction model. This calibration is done by digitizing the original survival curves of the two prediction models as published by *Margonis et al.* (expected outcomes, **darker lines**) and these curves are compared with the actual survival curves of the NCR cohort (observed outcomes, **lighter lines**). The risk table displays the number at risk for the NCR cohort.

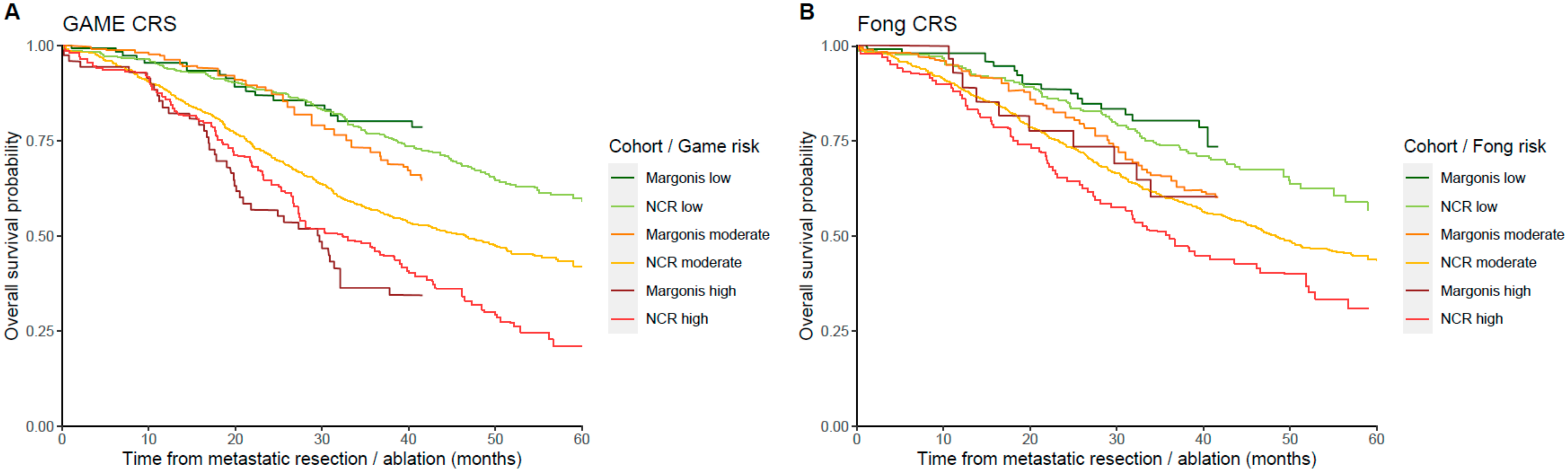

Number at risk (A)

low	335	322	302	272	218	140	66
moderate	661	600	510	419	330	205	89
high	110	101	80	56	43	25	7

Number at risk (B)

low	227	220	203	172	126	67	31
moderate	772	707	609	514	422	275	127
high	106	95	79	62	46	29	14

Supplementary Figure S3. A combined figure containing on the left, a contingency table showing the frequency distribution of patients among the risk categories (low, moderate, high) following the Fong and GAME prediction model, their corresponding 3-year survival rate estimate, which is also indicated by the heat map for each category. The corresponding survival curves for the groups are displayed in the KM plot on the right. Number of patients does not count up to total number of patients in study due to rounding effects of analyses in the imputed database.

	Fong low	Fong moderate	Fong high	Total
**GAME low**	*n*=154 (13.9%)<br>Survival: 79%	*n*=181 (16.4%)<br>Survival: 75%	*n*=0 (0.0%)<br>-	335
**GAME moderate**	*n*=71 (6.4%)<br>Survival: 64%	*n*=530 (47.9%)<br>Survival: 57%	*n*=60 (5.4%)<br>Survival: 47%	661
**GAME high**	*n*=3 (0.3%)	*n*=61 (5.5%)<br>Survival: 42%	*n*=46 (4.2%)<br>Survival: 54%	110
**Total**	228	772	106	1106

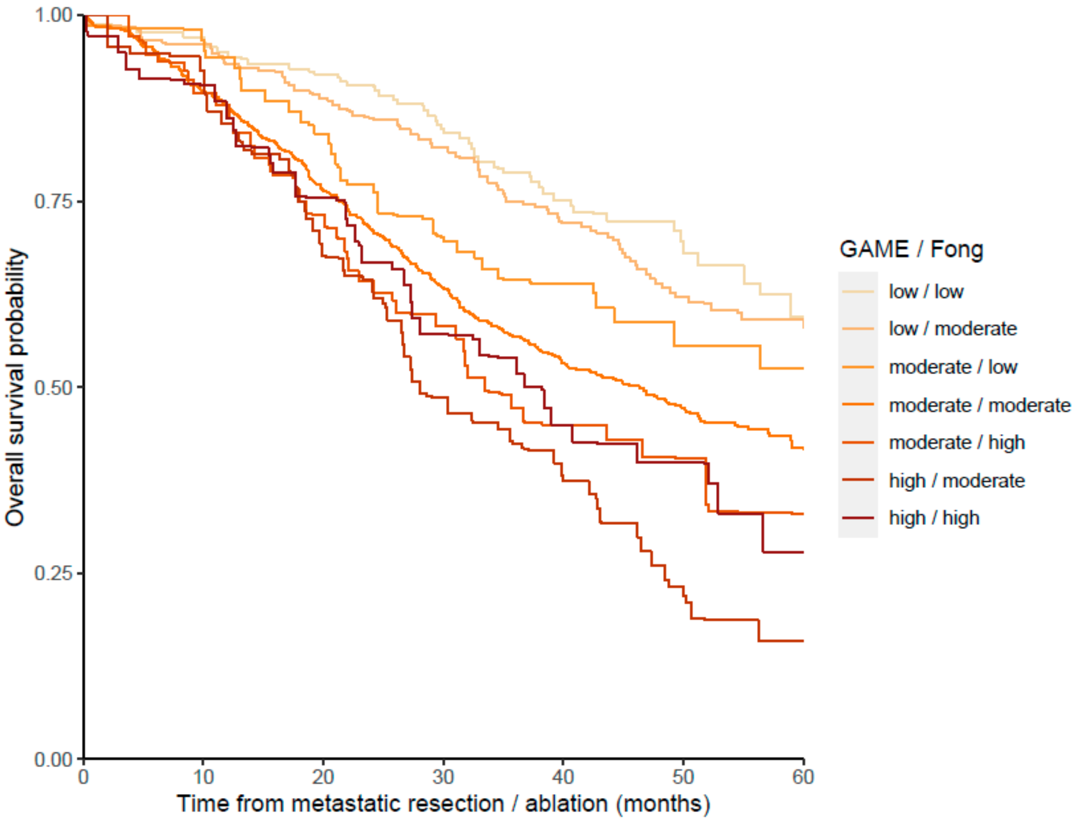

Number at risk

L / L	154	149	141	124	89	50	15
L / M	181	174	161	148	131	92	64
M / L	71	69	60	48	38	30	4
M / M	530	477	406	335	269	173	78
M / H	60	56	44	36	26	19	4
H / M	61	57	43	30	23	13	3
H / H	46	41	35	27	21	17	4

Supplementary Figure S4. Kaplan-Meier analysis showing disease-free survival (DFS) curves for GAME risk groups for patients without **(A)** and with **(B)** perioperative systemic therapy and for age groups ≤ 70 years **(E)** and > 70 years **(F)**. The DFS outcomes of Fong risk groups are shown for patients without **(C)** and with **(D)** perioperative systemic therapy and for age groups ≤ 70 years **(G)** and > 70 years **(H)**. Red lines represent the low risk groups, green lines the moderate risk and yellow lines the high risk group.

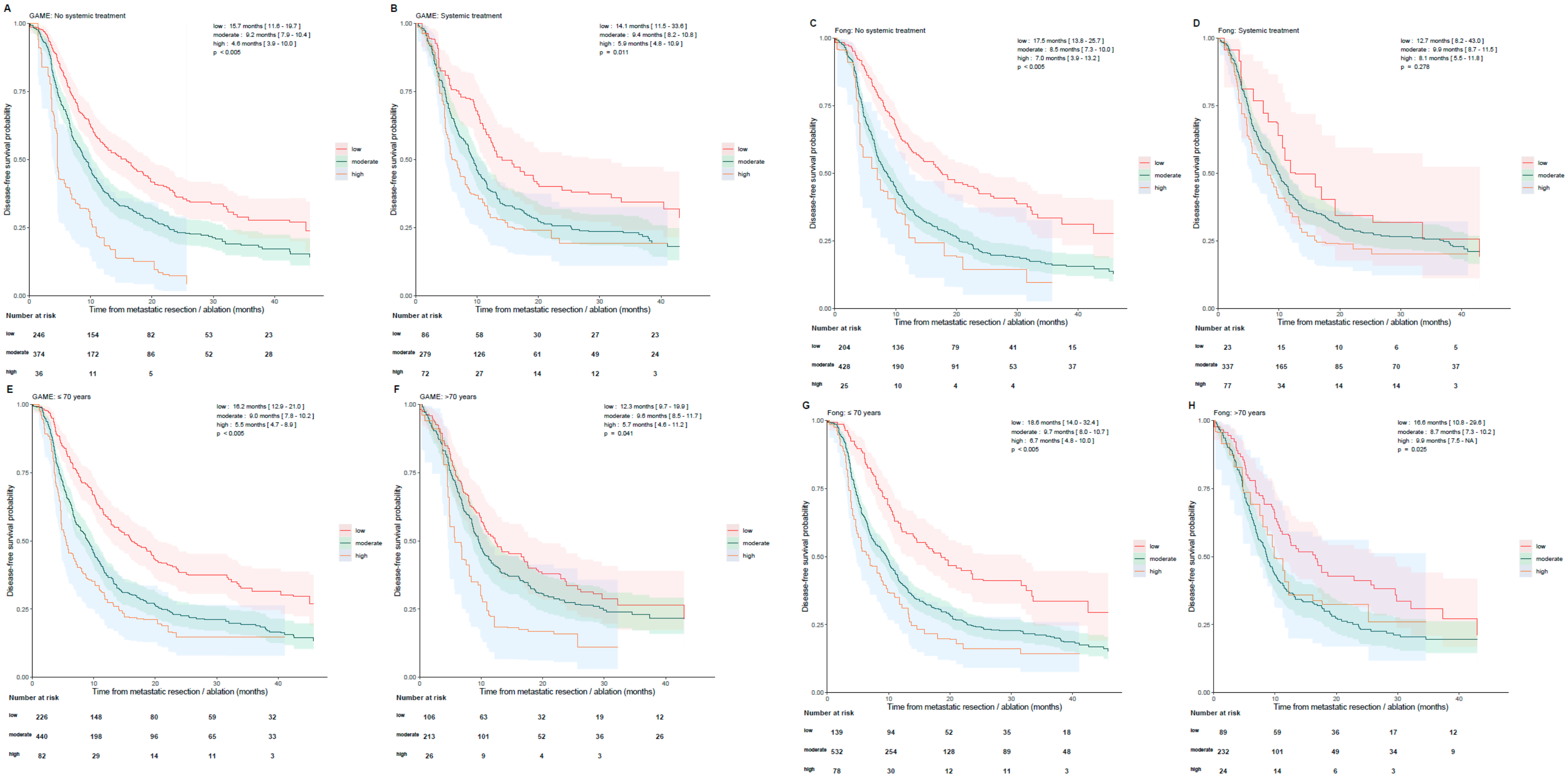

Supplementary Table S2. Pooled Harrell's concordance index with 95% confidence intervals for 1- and 3-year overall survival and disease-free survival outcomes for GAME and Fong risk scores in subgroups without and with perioperative systemic therapy. Survival estimates at 1- and 3- years for low, moderate and high risk groups according to GAME and Fong prediction model.

WITHOUT PERIOPERATIVE SYSTEMIC THERAPY								
	**GAME score**	**Survival estimates GAME risk categories**			**Fong score**	**Survival estimates Fong risk categories**		
	**C-index [95% C.I.]**	**Low (%)**	**Moderate (%)**	**High (%)**	**C-index [95% C.I.]**	**Low (%)**	**Moderate (%)**	**High (%)**
OS								
1-year OS	0.592 [0.518-0.667]	94	89	84	0.596 [0.532-0.661]	95	89	84
3-year OS	0.610 [0.574-0.647]	78	57	43	0.593 [0.559-0.628]	75	60	48
5-year OS	0.602 [0.569-0.635]	60	41	26	0.594 [0.563-0.624]	59	42	
DFS								
1-year DFS	0.584 [0.553-0.614]	56	39	21	0.606 [0.578-0.635]	61	37	31
3-year DFS	0.579 [0.551-0.606]	28	18	9	0.601 [0.575-0.627]	33	16	
**WITH PERIOPERATIVE SYSTEMIC THERAPY**								
	**GAME score**	**Survival estimates GAME risk categories**			**Fong score**	**Survival estimates Fong risk categories**		
	**C-index [95% C.I.]**	**Low (%)**	**Moderate (%)**	**High (%)**	**C-index [95% C.I.]**	**Low (%)**	**Moderate (%)**	**High (%)**
OS								
1-year OS	0.588 [0.511-0.664]	96	88	86	0.538 [0.460-0.617]	96	89	88
3-year OS	0.590 [0.549-0.631]	74	56	49	0.556 [0.515-0.598]	60	60	51
5-year OS	0.590 [0.554-0.627]	58	43	23	0.557 [0.519-0.594]		46	29

DFS								
1-year DFS	0.589 [0.551-0.626]	58	39	30	0.563 [0.528-0.598]	50	42	35
3-year DFS	0.581 [0.547-0.616]	34	23	19	0.559 [0.527-0.591]	26	26	20

When not indicated, the number of patients was too small to calculate the survival estimate.
Abbreviations; C-index, concordance index; DFS, disease-free survival; OS, overall survival.

Supplementary Table S3. Pooled Harrell's concordance index with 95% confidence intervals for 1- and 3-year overall survival and disease-free survival outcomes for GAME and Fong risk scores in subgroups of ≤ 70 years and > 70 years. Survival estimates at 1- and 3-years for low, moderate and high risk groups according to GAME and Fong prediction model.

AGE ≤ 70 YEARS								
	**GAME score**	**Survival estimates GAME risk categories**			**Fong score**	**Survival estimates Fong risk categories**		
	**C-index [95% C.I.]**	**Low (%)**	**Moderate (%)**	**High (%)**	**C-index [95% C.I.]**	**Low (%)**	**Moderate (%)**	**High (%)**
**OS**								
1-year	0.611 [0.540-0.681]	96	90	88	0.583 [0.515-0.650]	96	92	86
3-year	0.618 [0.585-0.652]	82	59	50	0.588 [0.555-0.621]	76	64	48
5-year	0.613 [0.585-0.642]	64	45	21	0.584 [0.554-0.613]	56	48	32
**DFS**								
1-year	0.601 [0.573-0.628]	59	38	29	0.595 [0.569-0.621]	61	40	32
3-year	0.594 [0.568-0.619]	32	19	15	0.591 [0.567-0.614]	34	21	14
**AGE > 70 YEARS**								
	**GAME score**	**Survival estimates GAME risk categories**			**Fong score**	**Survival estimates Fong risk categories**		
	**C-index [95% C.I.]**	**Low (%)**	**Moderate (%)**	**High (%)**	**C-index [95% C.I.]**	**Low (%)**	**Moderate (%)**	**High (%)**
**OS**								
1-year	0.572 [0.491-0.654]	90	85	79	0.601 [0.527-0.675]	93	83	89
3-year	0.580 [0.533-0.627]	67	52	37	0.590 [0.547-0.634]	70	50	58
5-year	0.575 [0.531-0.618]	49	36	37	0.589 [0.548-0.630]	58	34	
**DFS**								
1-year	0.554 [0.511-0.597]	51	41	22	0.584 [0.542-0.625]	58	37	41
3-year	0.547 [0.507-0.587]	26	23	22	0.575 [0.536-0.613]	31	20	

When not indicated, the number of patients was too small to calculate the survival estimate.
Abbreviations; C-index, concordance index; DFS, disease-free survival; OS, overall survival.
